# Inhibitory Effect of the* Punica granatum* Fruit Extract on Angiotensin-II Type I Receptor and Thromboxane B2 in Endothelial Cells Induced by Plasma from Preeclamptic Patients

**DOI:** 10.1155/2016/6028989

**Published:** 2016-02-18

**Authors:** Widya Kusumawati, Kusnarman Keman, Setyawati Soeharto

**Affiliations:** ^1^Dharma Husada Midwifery Academy, Jalan Penanggungan No. 41, Kelurahan Lirboyo, Kecamatan Mojoroto, Kediri, East Java 64117, Indonesia; ^2^Midwifery Master Study Programme, Faculty of Medicine, Brawijaya University, Jalan Veteran, Kecamatan Lowokwaru, Malang, East Java 65145, Indonesia; ^3^Obstetric and Ginecology Laboratory, Saiful Anwar General Hospital, Faculty of Medicine, Brawijaya University, Jalan Jaksa Agung Suprapto No. 2, Malang, East Java 65122, Indonesia; ^4^Pharmacology Laboratory, Faculty of Medicine, Brawijaya University, Jalan Veteran, Kecamatan Lowokwaru, Malang, East Java 65145, Indonesia

## Abstract

This study aims to evaluate whether the* Punica granatum* fruit extract modulates the Angiotensin-II Type I receptor (AT1-R) and thromboxane B2 level in endothelial cells induced by plasma from preeclamptic patients. Endothelial cells were obtained from human umbilical vascular endothelial cells. At confluence, endothelial cells were divided into five groups, which included endothelial cells exposed to 2% plasma from normal pregnancy (NP), endothelial cells exposed to 2% plasma from preeclamptic patients (PP), and endothelial cells exposed to PP in the presence of ethanolic extract of* Punica granatum *(PP + PG) at the following three doses: 14; 28; and 56 ppm. The expression of AT1-R was observed by immunohistochemistry technique, and thromboxane B2 level was done by immunoassay technique. Plasma from PP significantly increased AT1-R expression and thromboxane B2 levels compared to cells treated by normal pregnancy plasma. The increasing of AT1-R expression significantly (*P* < 0.05) attenuated by high dose treatments of* Punica granatum* extract. Moreover, the increasing of thromboxane B2 levels significantly (*P* < 0.05) attenuated by lowest dose treatments of* Punica granatum* extract. We further concluded that* Punica granatum* fruit protects and inhibits the sensitivity of endothelial cells to plasma from preeclamptic patients due to inhibition of AT1-R expression (56 ppm) and reduced thromboxane B2 levels (14 ppm).

## 1. Introduction

Preeclampsia is a pregnancy-associated disorder characterized by collective symptoms such as increasing blood pressure and proteinuria. This complex syndrome leads to maternal and fetal morbidity and mortality. The pathophysiological mechanism of this syndrome remains unclear. Predisposing demographic, genetic, and environmental risk factors was involved in the defect of the placenta. Defective placentation and improper trophoblast invasion of the myometrium cause reduction in uteroplacental perfusion pressure which stimulates the release of complex circulating bioactive factors [1–5].

The dysfunction of the vascular system found in preeclampsia is associated with the production of vasoactive factors by endothelial cells. One pathway which is involved in this dysfunction is the renin angiotensin aldosterone system. A low sensitivity to the vasoconstrictor angiotensin-II (Ang-II) is found in normal pregnancy, but this sensitivity increases in preeclampsia case prior to the clinical onset of disease [6–9]. Ang-II exerts its effects via two receptors and its binding to the Ang-II Type I receptor (AT1-R) induces contraction [7].

Preeclampsia is also associated with disproportional of serum factors, including thromboxane and prostacyclin with undefined mechanism of this release. The thromboxane levels are found to significantly increase only in severe preeclampsia. The ratio of thromboxane greatly increases in severe cases [10, 11]. Besides, the serum was obtained from preeclampsia which also contains cytotoxic factors that can damage endothelial cells. So this pathomechanism must diminish or be inhibited by drug or other pharmacological agents.* Punica granatum* is an Indonesian ancient fruit, the main compounds of which are polyphenols and carbohydrates [12] that may act as an alternative herbs therapy in preeclampsia treatment [13–16]. As far as we know, there was no previous study which evaluated the effect of* Punica granatum *on AT1-R and thromboxane B2 level of endothelial induced by preeclamptic plasma, so this pathomechanism must diminish or be inhibited by drug or other pharmacological agents. Therefore, this study is aimed at investigating whether* Punica granatum* fruit extracts modulate the AT1-R and thromboxane B2 level in endothelial cells challenged by plasma from preeclamptic patients.

## 2. Material and Methods

### 2.1. Endothelial Cells Isolation and Culture

The endothelial cells were collected from the human umbilical vein. This umbilical vein was obtained from pregnant mother with the following characteristics: a healthy and at term (38 weeks of gestation) pregnancy, hemoglobin level ≥ 10 g/dL, having performed a section cesarean delivery. Immediately postpartum, 10 cm of the umbilical cord was inserted in buffer (100 mL Hank's Balance Salt Solution (HBSS), gentamycin (GENTA, MERCK, Germany), sodium hydrogen bicarbonate, 4 mL red phenol, 2 mL HEPES solution, and deionized water) and kept cold in the transportation process to target laboratory. In order to get the best result, the isolation was performed under 12 hours after umbilical cord collection [17].

When the umbilical cord was in clean condition, a cannula was inserted (±1.5 cm) at one edge of the vein and secured tightly using suture. After that, the veins were gently washed with PBS and then closed off tightly at the distal edge to the cannula. After washing, the collagenase (SIGMA, type HA, C-6885) (5 mg/10 mL; 10 mL) was injected into the vein by 10 mL syringe then incubated at 37°C for 8 minutes. The cell pellet was suspended in 4 mL 199 culture medium (SIGMA, M-5017, USA) which was supplemented with a cocktail (gentamycin, bicarbonate phenol red, 20% fetal bovine serum (GIBCO), and 20 mL new born calf serum (SIGMA, N-4637, USA). This cell suspension was cultivated into wells that had been coated with gelatin (SIGMA, G1393). Cells were allowed to achieve the confluency at 37°C and 5% CO_2_.

### 2.2. Isolation of Plasma Preeclamptic Patients

For blood collection, normal pregnancy and preeclamptic patients all gave informed consent. Written informed consent should be completed by Ethics Committee of Health Research, Medical Faculty, Brawijaya University, Malang, East Java, Indonesia. The clinical signs of preeclamptic patients were systolic blood pressure (≥160 mmHg) and diastolic blood pressure (≥110 mmHg) in two measurements (4–6 hours between measurements) and proteinuria (≥2+) on dipstick. Plasma was isolated from 2 mL of whole blood in citrate anticoagulant. The blood was centrifuged at 1000 g, for 10 minutes at −40°C. Plasma was used in cell culture experiments as detailed in the following sections [17].

### 2.3. Cell Treatment

Once confluent, cells treatments were divided into 5 replicated experiments including endothelial cells exposed to 2% plasma from normal pregnancy (NP); cells treated with 2% plasma preeclamptic patients (PP); PP + the ethanolic extract from* Punica granatum* (PG) fruit at several doses, including 14 ppm; 28 ppm; and 56 ppm.

### 2.4. *Punica granatum* Extraction

*Punica granatum*, in dry condition was obtained from Bandungan village, Semarang, Central Java, Indonesia. The extraction was initiated by grinding the meat of the* Punica granatum *fruit using 400 mesh grinder. Subsequently, maceration was performed on 100 gram of powder (70% ethanol (1 : 3 ratio) for 2 × 24 hours). After that, polyphenolic extract was separated from the* Punica granatum* sedimentation. This polyphenolic extract was dried by vacuum oven for 8-9 hours at 45–50°C. This procedure yields 25 grams of* Punica granatum* extract.

### 2.5. Analysis of AT1-R Expression

AT1-R expression of endothelial cells was measured by immunohistochemistry technique. The details of the methods were explained formerly [18]. We used Rabbit Anti-Angiotensin-II Type 1 Receptor Polyclonal Antibody (Bioss Antibodies, Catalog bs-0630R, Woburn, Massachusetts, USA).

### 2.6. Analysis of Thromboxane B2 Levels

The levels of thromboxane B2 were determined using ELISA technique (R&D system; catalog KGE011, Minneapolis, MN, USA). The procedure was performed according to the instructions from the manufacturer.

### 2.7. Statistical Analysis

Data are presented as mean ± standard deviation and differences between groups were analyzed using one-way analysis of variance using SPSS 17.0 statistical package software. The post hoc test was used to examine significant differences among treatments (*P* < 0.05 was considered statistically significant).

## 3. Results

**Figure 1:**
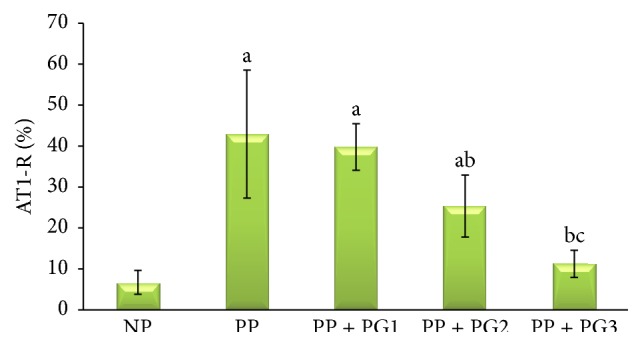
The endothelial expression of Angiotensin-II Type I receptor was measured by immunohistochemistry technique. The data represents 5 replicated experiments (mean value ± SD), ^a^ *P* < 0.05 in comparison with endothelial cells exposed to 2% plasma from normal pregnancy (NP) group; ^b^ *P* < 0.05 in comparison with endothelial cells exposed to 2% plasma from preeclamptic patients (PP) group; ^c^ *P* < 0.05 in comparison with first dose (14 ppm)* Punica granatum* group.

**Figure 2:**
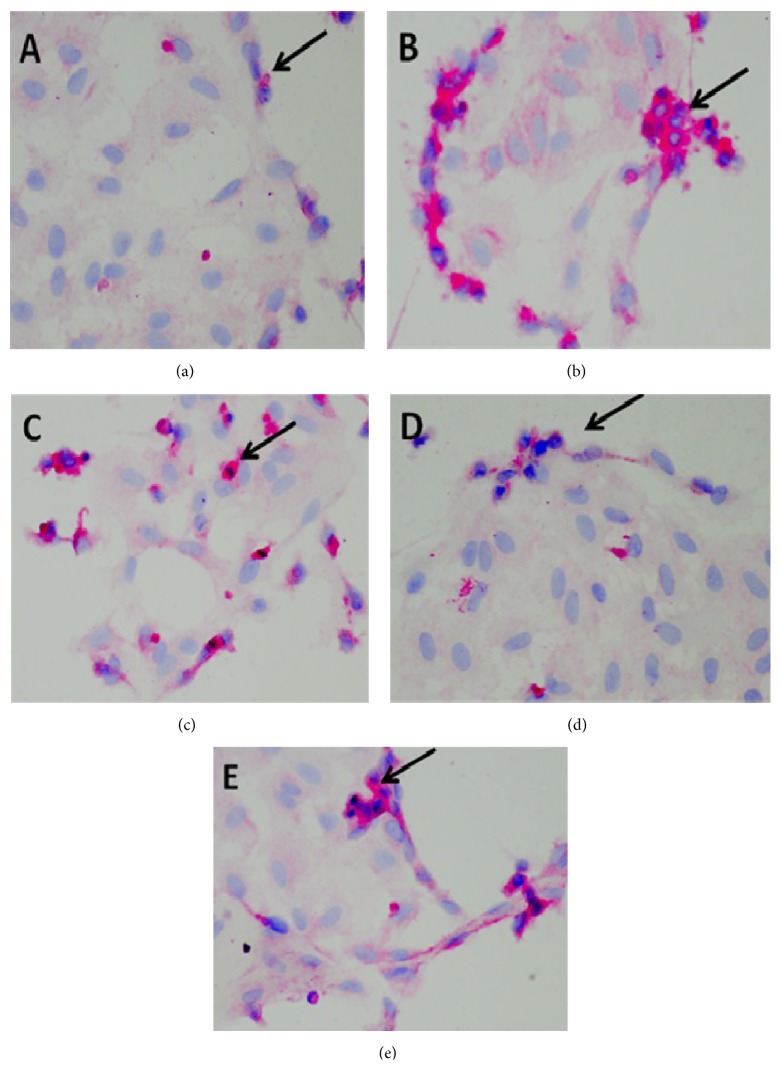
Representative micrograph of Angiotensin-II Type I receptor (black arrow) in endothelial cells induced by plasma preeclamptic patients. Endothelial cells exposed to 2% plasma from normal pregnancy (a), endothelial cells exposed to 2% plasma from preeclamptic patients (b), and endothelial cells exposed to PP in the presence of ethanolic extract of* Punica granatum *(PP + PG) at the following three doses: 14 (c); 28 (d); and 56 ppm (e).

Table 1 and Figures 1 and 2 present the AT1-R expression in the endothelial cells from each group. The AT1-R expression is significantly higher in the PP group compared to the NP group (*P* < 0.05). The highest dose of the* Punica granatum* fruit extract significantly prevented PP-induced increase in AT1-R expression (*P* < 0.05) reaching a similar level with the NP group (*P* > 0.05).

**Figure 3:**
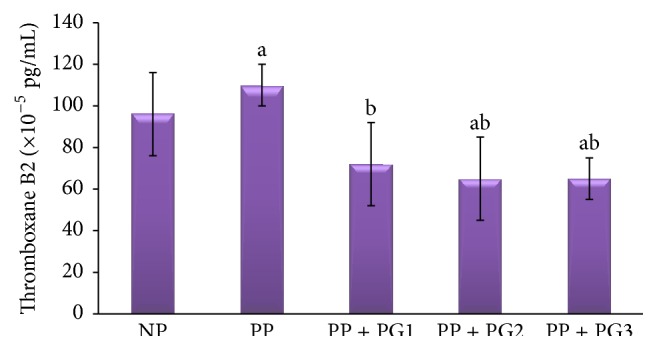
The level of thromboxane B2 in endothelial cells of each experimental group. Values are presented as mean ± SD; ^a^ *P* < 0.05 in comparison with endothelial cells exposed to 2% plasma from normal pregnancy (NP) group; ^b^ *P* < 0.05 in comparison with endothelial cells exposed to 2% plasma from preeclamptic patients (PP) group; PP + G1: first-dose* Punica granatum* administered group; PP + G2: second-dose* Punica granatum* administered group; PP + G3: third-dose* Punica granatum* administered group; pg/mL: picogram/milliliter.

Table 2 and Figure 3 present the thromboxane B2 level in the medium of endothelial cells from each experimental group. The thromboxane B2 levels were significantly greater in the PP group compared to the NP group (*P* < 0.05). Out of 14 ppm, 28 ppm, and 56 ppm of extract, only lowest dose of the* Punica granatum* fruit extract significantly prevented PP-induced increase in thromboxane B2 levels (*P* < 0.05) reaching a similar level with the NP group (*P* > 0.05).

## 4. Discussion

Women with preeclampsia, on the other hand, did not show this resistance to angiotensin-II, which can already be observed as early as week 10 of gestation and thus well before the onset of clinically apparent symptoms. This increased sensitivity was still present 8 months after pregnancy [19]. There are several explanations for this increase in sensitivity. First, the adipose tissue of patients with preeclampsia displays elevated AT1-R. Increased AT1-R expression may also be present in other tissues [20]. In this study, this is the first report which found that the AT1-R expression was significantly higher in the PP group compared to the NP group (*P* < 0.05). This study showed that endothelial cell modulates the AT1-R expression coming from exposure to preeclamptic plasma. The highest dose of the* Punica granatum* fruit extracts significantly prevented PP-induced increase in AT1-R expression (*P* < 0.05) reaching a similar level with control (NP) group (*P* > 0.05). Our finding indicated that the* Punica granatum *fruit extract inhibits the signal for the AT1-R expression of endothelial cells.

In this study, the thromboxane B2 level is significantly greater in the PP group compared to the NP group (*P* < 0.05). This finding indicates that plasma from preeclamptic patient contains bioactive factor for activating the cyclooxygenase enzyme. The phospholipase A2 activity acts to catalyze the hydrolysis of membrane phospholipids sn-2 ester bond to produce an arachidonic acid, which is the substrate of cyclooxygenase. This enzyme will convert the arachidonic acid into thromboxane. In fact, elevated maternal levels of soluble phospholipase A2 and arachidonic acid have been reported in women with preeclampsia [21, 22]. Besides, the trophoblast cells from preeclamptic placentas secreted higher level of phospholipase A2 compared with the normal placentas. This increase of phospholipase A2 activity is positively correlated to the greater levels of thromboxane [22, 23]. Out of 14 ppm, 28 ppm, and 56 ppm of extract, only lowest dose of the* Punica granatum* fruit extract significantly prevented PP-induced increase in thromboxane B2 level (*P* < 0.05) reaching a similar level with NP group (*P* > 0.05). We hypothesized that* Punica granatum *fruit extract inhibits the cyclooxygenase enzyme for thromboxane production. Several reports showed that* Punica granatum *fruit extract acts as cyclooxygenase inhibitors [24, 25].

In conclusion,* Punica granatum* fruit extract protects and inhibits the sensitivity of endothelial cells to plasma from preeclamptic patients due to inhibition of AT1-R expression (56 ppm) and reduced thromboxane B2 levels (14 ppm).

## Figures and Tables

**Table 1:** Level of Angiotensin-II Type I receptor in endothelial cells induced by plasma preeclamptic patients.

Expression	NP	PP	PP + *Punica granatum*		
			14 ppm	28 ppm	56 ppm
AT1-R (% cells)	6.72 ± 2.91	42.19 ± 15.63^a^	39.78 ± 5.67^a^	25.33 ± 7.57^ab^	11.25 ± 3.31^bc^

Note: values are presented as mean ± SD; ^a^ *P* < 0.05 in comparison with NP group; ^b^ *P* < 0.05 in comparison with PP group; ^c^ *P* < 0.05 in comparison with first dose (14 ppm) *Punica granatum* group; NP: plasma from normal pregnancy; PP: plasma from preeclamptic patients; ppm: part per million.

**Table 2:** Thromboxane B2 level in endothelial cells induced by plasma preeclamptic patients.

Level	NP	PP	PP + *Punica granatum*		
			14 ppm	28 ppm	56 ppm
TXB_2_ (pg/mL)	0.00096 ± 0.0002	0.00110 ± 0.0001^a^	0.00072 ± 0.0002^b^	0.00065 ± 0.0002^ab^	0.00065 ± 0.0001^ab^

Note: values are presented as mean ± SD; TXB_2_: thromboxane B2; ^a^ *P* < 0.05 in comparison with NP group; ^b^ *P* < 0.05 in comparison with PP group; ppm: part per million; pg/mL: picogram/milliliter.
